# Profile and prevalence of hearing complaints in the elderly^☆^

**DOI:** 10.1016/j.bjorl.2016.06.015

**Published:** 2016-07-31

**Authors:** Magda Aline Bauer, Ângela Kemel Zanella, Irênio Gomes Filho, Geraldo de Carli, Adriane Ribeiro Teixeira, Ângelo José Gonçalves Bós

**Affiliations:** a Pontifícia Universidade Católica do Rio Grande do Sul (PUC-RS), Programa de Pós-Graduação em Gerontologia Biomédica, Porto Alegre, RS, Brazil; b Universidade Federal do Rio Grande do Sul (UFRGS), Porto Alegre, RS, Brazil; c Pontifícia Universidade Católica do Rio Grande do Sul (PUC-RS), Porto Alegre, RS, Brazil

**Keywords:** Hearing loss, Epidemiologic studies, Aged, Perda auditiva, Estudo epidemiológico, Idoso

## Abstract

**Introduction:**

Hearing is essential for the processing of acoustic information and the understanding of speech signals. Hearing loss may be associated with cognitive decline, depression and reduced functionality.

**Objective:**

To analyze the prevalence of hearing complaints in elderly individuals from Rio Grande do Sul and describe the profile of the study participants with and without hearing complaints.

**Methods:**

7315 elderly individuals interviewed in their homes, in 59 cities in the state of Rio Grande do Sul, Brazil, participated in the study. Inclusion criteria were age 60 years or older and answering the question on auditory self-perception. For statistical purposes, the chi-square test and logistic regression were performed to assess the correlations between variables.

**Results:**

139 elderly individuals who did not answer the question on auditory self-perception and 9 who self-reported hearing loss were excluded, totaling 7167 elderly participants. Hearing loss complaint rate was 28% (2011) among the elderly, showing differences between genders, ethnicity, income, and social participation. The mean age of the elderly without hearing complaints was 69.44 (±6.91) and among those with complaint, 72.8 (±7.75) years. Elderly individuals without hearing complaints had 5.10 (±3.78) years of formal education compared to 4.48 (±3.49) years among those who had complaints. Multiple logistic regression observed that protective factors for hearing complaints were: higher level of schooling, contributing to the family income and having received health care in the last six months. Risk factors for hearing complaints were: older age, male gender, experiencing difficulty in leaving home and carrying out social activities.

**Conclusions:**

Among the elderly population of the state of Rio Grande do Sul, the prevalence of hearing complaints reached 28%. The complaint is more often present in elderly men who did not participate in the generation of family income, who did not receive health care, performed social and community activities, had a lower level of schooling and were older.

## Resumo

**Introdução:**

A audição é essencial para o processamento de eventos acústicos e emissão e compreensão dos sinais de fala. A perda auditiva pode estar associada ao declínio cognitivo, depressão e redução da funcionalidade.

**Objetivo:**

Analisar a prevalência de queixa auditiva em idosos do Rio Grande do Sul e descrever o perfil dos participantes com e sem queixa auditiva.

**Método:**

Participaram do estudo 7.315 idosos entrevistados em suas residências, em 59 cidades gaúchas. Os critérios de inclusão adotados foram ter 60 anos ou mais de idade e terem respondido à questão sobre autopercepção auditiva. Para fins estatísticos foi realizado o teste Qui-quadrado e regressão logística para avaliar as correlações entre as variáveis.

**Resultados:**

Foram excluídos 139 idosos sem resposta à autopercepção auditiva e nove por autorreferirem surdez (7.167 participantes). A frequência de queixa de perda auditiva foi de 28% (2011) dos idosos, apresentando diferença entre gêneros, etnia, renda, participação social. A média de idade dos idosos sem queixa auditiva foi de 69,44 (±6,91) e com queixa 72,8 (±7,75) anos. Os idosos sem queixa auditiva apresentaram 5,10 (±3,78) anos de estudo comparado a 4,48 (±3,49) anos dos com queixa. A regressão logística múltipla observou que foram fatores protetores para a queixa auditiva maior escolaridade, contribuir na renda familiar e ter recebido atendimento de saúde nos últimos seis meses. Fatores de risco para a queixa auditiva foram idade mais avançada, sexo masculino, apresentar dificuldade de sair de casa e realizar atividades sociais.

**Conclusões:**

Na população idosa do Rio Grande do Sul a prevalência de queixa auditiva atingiu 28%. A queixa está mais presente em idosos homens, sem participação na renda familiar, não receberam atendimento de saúde, tinham atividade social e comunitária, com menor escolaridade e maior idade.

## Introduction

Hearing is essential for the processing of acoustic information and for the production and understanding of speech signals. The consequences of hearing loss vary according to its type, degree and age of onset. In adults and elderly individuals, one generally observes isolation, with diminished participation in social and family life, sometimes due to the fear of becoming the target of ridicule or contempt.^1^ Hearing loss can also be associated with cognitive decline, depression and reduced functional status.^2^

Because of the increase in the numbers of the elderly, it is appropriate to understand the factors related to aging and frailty, especially factors related to becoming incapacitated.^3^ These can be characterized by the interaction between the individual's dysfunction (organic and/or structural), restrictions in social participation and environmental factors that may interfere with the performance of individual activities.^4^

Therefore, it is important to evaluate the functional capacity of the elderly in order to correlate it with the practical aspects of personal care in the maintenance and performance of the basic and complex activities of daily living.^5^ Among the factors to be assessed is hearing, which is one of the major sensory alterations^6^ that can change the daily habits of the elderly.

Hearing loss diagnosis and rehabilitation should be carried out early after recognition, regardless of the individual's age. However, in many cases the hearing loss occurs gradually, with a slow progression that is not noticed or is neglected.^1^ Thus, hearing screening should be a standard procedure, aiming at the early diagnosis to avoid the adverse effects of auditory deprivation.^7^ Previous studies have indicated that the complaint of difficulty hearing may be a good predictor of an existing loss,^8^ with a greater sensitivity and specificity of the predictive value of the self-reported hearing loss in elderly individuals compared to other ages.^9^

Despite the relevance of hearing loss in the elderly, few studies have been carried out or been able to determine its incidence in the Brazilian population or in their states. One existing study carried out in São Paulo showed a prevalence of 30% of hearing loss in the elderly population.^2^ This information is important so that the adequate hearing health care measures can be taken and the magnitude of this issue can be assessed in the population.

Therefore, this study aims to analyze the prevalence of self-reported hearing complaints in elderly individuals from the state of Rio Grande do Sul, Brazil and to describe the epidemiological factors associated with elderly individuals with and without hearing complaints.

## Methods

This was characterized as a descriptive and cross-sectional study, which is part of a larger survey, with focus on hearing function of the elderly. The questionnaire was inspired by the Global Age-friendly Cities: A Guide, of the World Health Organization. The analyzed and discussed data were collected between the years 2010 and 2011.

In total, 7315 elderly individuals were interviewed in their homes, randomly selected from census sectors of 59 cities in the state of RS. Inclusion criteria were age of 60 years or older and attending the interview. Participants who could not answer the question on auditory self-perception due to cognitive and communication impairment were excluded. All participants or their guardians signed the free and informed consent form. The project was approved by the ethics committees of the institutions involved in the study (09/04931 and 481/09).

Among other issues the elderly were interviewed, about hearing perception. The variables gender (male and female), marital status (married, single, widowed, separated or divorced, did not know how to answer), ethnicity (White, Mixed-Race, Black, other), participation in family income (no income, main or sole provider, shared responsibility), received care for health problems in the last 6 months (received, did not receive), performs social (does, does not) or community activities (does, does not) and reports difficulties going out due to communication problems (has difficulties, has no difficulties) in addition to hearing aid use (uses, does not use) were treated as categorical variables and expressed as frequencies. Age and educational level (years of formal schooling), these were treated as numerical variables and expressed as mean and standard deviation. The question about hearing self-perception had the following response options: “excellent”; “good”; “regular”; “poor” and “very poor” – previously established in the questionnaire.

For the statistical analysis, auditory self-perception levels of good or excellent were grouped together, as were without complaint and regular, and poor or very poor. The chi-square test was used to test the association between hearing complaints and the other variables. Multiple logistic regression was used to calculate the odds ratio of hearing loss complaint being influenced by the assessed variables. Significance levels lower than 5% were considered statistically significant and between 5% and 10%, as indicative of significance.^10^

## Results

Of the 7315 participants, 139 were excluded because they did not answer the question about auditory self-perception (cognitive impairment) and 9 because they reported hearing loss, leaving 7167 elderly individuals in the sample (Table 1). The results were expressed as mean and standard deviation (SD). The mean age of the elderly without hearing complaints was 69.4 (6.91) and in those with complaint, 72.8 (7.75) years. The elderly without hearing complaints had on average 5.1 (3.79) years of education, which was greater than the 4.5 (3.49) years of education of those with complaints. Both age and educational level were significantly different between those with and without hearing complaints.

**Table 1:** Characteristics of the participants according to the level of hearing loss.

	Hearing complaint			*p*
	Without complaint	With complaint	Total	
*Sample*	5156 (71.94%)	2011 (28.06%)	7167	
Age (mean ± SD)	69.4 ± 6.91	72.8 ± 7.75	70.4 ± 7.32	<0.0001
Years of schooling (mean ± SD)	5.1 ± 3.79	4.5 ± 3.50	4.9 ± 3.72	<0.0001

*Gender*				0.0016
Female	2731(73.6%)	982 (26.4%)	3713 (51.8%)	
Male	2425 (70.2%)	1029 (29.8%)	3454 (48.2%)	

*Marital status*				<0.0001
Married	2341 (73.5%)	843 (26.5%)	3184 (44.4%)	
Single	256 (66.0%)	132 (34.0%)	388 (5.4%)	
Widowed	1599 (68.3%)	742 (31.7%)	2341 (32.7%)	
Separated/divorced	759 (75.9%)	241 (24.1%)	1000 (14%)	
Did not know how to answer	201 (79.1%)	53 (20.9%)	254 (3.5%)	

*Ethnicity*				0.0899
White	3578 (72.7%)	1341 (27.3%)	4919 (68.6%)	
Mixed-race	768 (70.3%)	325 (29.7%)	1093 (15.3%)	
Black	536 (69.0%)	241 (31.0%)	777 (10.8%)	
Others	248 (71.3%)	100 (28.7%)	348 (4.9%)	

*Income participation*				<0.0001
No participation	345 (68.4%)	159 (31.5%)	504 (7%)	
Main provider or only one responsible for income	2357 (69.7%)	1025 (30.3%)	3382 (47.2%)	
Shares responsibility	2454 (74.8%)	827 (25.2%)	3281 (45.8%)	

*Received care*				<0.0001
Did not receive	2972 (68.6%)	1360 (31.4%)	4332 (60.4%)	
Received	2178 (77.0%)	651 (23.0%)	2829 (39.5%)	

*Social activity*				0.0013
Does not perform	3066 (73.4%)	1112 (26.6%)	4178 (58.3%)	
Performs	2090 (69.9%)	899 (30.1%)	2989 (41.7%)	

*Community activity*				
Does not perform	4313 (73.3%)	1568 (26.7%)	5881 (82.1%)	<0.0001
Performs	843 (65.5%)	443 (34.4%)	1286 (17.9%)	

*Going out/communication difficulty*				0.0027
No difficulty	5127 (72.1%)	1986 (27.9%)	7113 (99.2%)	
Has difficulty	29 (53.7%)	25 (46.3%)	54 (0.8%)	

*Hearing aid use*				<0.0001
Does not use	5077 (73.3%)	1851 (26.7%)	6928 (96.7%)	
Uses	79 (33.1%)	160 (66.9%)	239 (3.3%)	

At the logistic regression analysis, both age and educational level maintained almost the same level of significance. Regarding the years of study, the results showed that participants with hearing complaints had fewer years of study. Each extra year of study was related to 3% lower chance of hearing complaints. In relation to age, each extra year was related to a 6% higher chance of hearing complaints (Table 2).

**Table 2:** Results of multiple logistic regression for the chance of having hearing loss complaints.

	Odds ratio	Confidence interval		*p*
Years of schooling	0.9690	0.9538	0.9844	0.0001
Age	1.0602	1.0519	1.0685	<0.0001
Gender (male/female)	1.1939	1.0640	1.3396	0.0026
Marital status (single/married)	1.4076	1.1056	1.7922	0.0055
Marital status (widowed/married)	0.9798	0.8546	1.1232	0.7695
Marital status (separated/married)	0.9690	0.8139	1.1536	0.7235
Income participation (main provider/no participation)	0.8903	0.7154	1.1080	0.2978
Income participation (shares/does not share)	0.7691	0.6175	0.9581	0.0192
Health care (yes/no)	0.7388	0.6577	0.8298	<0.0001
Community activity (yes/no)	1.4462	1.2434	1.6820	<0.0001
Difficulty going out due to communication problems (yes/no)	1.9518	1.0887	3.4993	0.0247
Social activity (yes/no)	1.1593	1.0253	1.3107	0.0183

The hearing complaints were significantly more frequent in men (*p* = 0.0016). The association between gender and hearing complaints remained significant in the multivariate analysis, after adjusting for other variables. Although the difference in the frequencies found in men and women with complaints was only 3.3% (Table 1), at the multiple analysis men had a 19% higher chance of having hearing complaints than women, after adjusting for other variables (Table 2). Regarding the marital status variable, it was significant for hearing loss complaints (*p* < 0.0001). Single and widowed individuals had the highest frequencies of hearing complaints, with the lowest being observed in married and separated individuals.

To better understand the association between marital status and hearing complaints, a simple logistic regression was performed, shown in Table 3. Widowers had a 29% higher chance of having hearing complaints, and in single individuals this percentage was 43%, compared to married individuals. In the multivariate analysis, after adjusting for other factors, the chance of widowed individuals having hearing complaints compared to married ones became non-significant, whereas the chance of singles having this complaint remained similar (41%) and significant, as shown in Table 2. Therefore, the observed difference between married and widowed individuals in relation to hearing complaints is dependent on other variables, including age.

**Table 3:** Result of the simple logistic regression of marital status for the chance of having hearing loss complaints in comparison to the married marital status.

	Odds ratio	Confidence interval	*p*
Marital status (single/married)	1.4319	1.1442 ± 1.7919	0.0017
Marital status (widowed/married)	1.2886	1.1459 ± 1.4491	<0.0001
Marital status (separated/married)	0.8818	0.7477 ± 1.0399	0.1348

As for income, only 7% of the sample reported having no participation in family income (Table 1). These individuals showed higher frequency of hearing complaints. Those who participated or were the only or main providers of family income had a significantly lower chance of having hearing complaints (Table 2).

Most of the elderly had not received health care in the six months before the interview (60.4%) (Table 1). However, the individuals who did receive health care had a significantly lower chance of listing hearing complaints (26%). It was found that having received health care in the last 6 months significantly lowered the chance of having hearing complaints compared to the elderly who did not receive care. Moreover, regarding follow-up, only four elderly individuals reported having received audiological follow-up.

Most participants did not engage in social or community activities, but both activities were significantly correlated with hearing complaints both in the simple analysis and after adjusting for other variables. Those who attended such activities showed higher frequency of complaints when compared to those who did not participate. This can be explained considering that individuals who are exposed to some activities can perceive their worse hearing performance than others who are not exposed to them, i.e., at home or in a familiar environment that adapts to them.

People who reported not leaving the house due to communication difficulties comprised only 0.8% of the sample (Table 1), but this factor was a very important one, as almost 50% of them had hearing complaints. People who did not leave the house due to communication difficulties had a 95% higher chance of having hearing complaints (Table 2).

Of 2011 elderly individuals who reported hearing complaints, only 8% used a hearing aid (Table 1). Most of them, 67% of those who used a hearing aid, had hearing complaints. However, 33% did not have complaints.

## Discussion

The incidence of self-reported hearing loss in our sample of elderly individuals aged 60 years and older was 28%, similar to another population-based study carried out in São Paulo, which showed a prevalence of 30%.^2^ Also similar to that study, the present one was based on the subjective complaint of hearing difficulty. We believe that the observed frequency would be higher if these elderly had undergone hearing assessment through audiometry. A previous study performed a comparison between hearing complaints and hearing loss and found that the latter was more frequent,; of the 50 elderly individuals in the sample, only 12 (24%) had a specific complaint of hearing loss, although 33 (66%) had mild, moderate, severe or profound hearing loss.^11^ Other studies using audiometry also found higher figures. A study by Mattos and Veras^12^ carried out with participants from a university extension project with the elderly, pointed out a prevalence of 41% of hearing loss complaint. The study by Costi et al.^13^ observed a 45% prevalence of hearing complaints among participants from a group of senior citizens. The differences can be explained by the study design, the place where the study was carried out and the individuals who comprised the sample.

Population-based studies on hearing loss are not common, either in Brazil or in other countries. Only a few studies were found on this topic. One of them, a literature review that pooled samples from European countries with individuals aged 60 years or older, found that approximately 30% of men and 20% of women had some degree of hearing loss at 70 years of age, as well as 55% of men and 45% of women at age 80.^14^ This study also found a higher prevalence of hearing complaints among the oldest old, with males being the most often affected. The increase in the complaints of hearing loss due to age can be explained by the fact that presbycusis is progressive and increases age,^15^ which would lead to a higher number of individuals with self-reported hearing complaints.

The influence of years of schooling can be observed in the assessed data, as the higher the educational level, the lower the chance of having a hearing loss complaint. A similar result was observed in studies in both Brazilian^2^ and European elderly^16^ and it might mean that more years of schooling result in greater care with one's health and the adoption of preventive measures to preserve hearing. Our data also suggest that income can interfere with hearing care, as income was a significantly protective factor for hearing complaints.

When assessing the question about receiving health care and having hearing loss, it was observed that the elderly who received such care had fewer complaints. It is believed that such occurrence is due to the fact that individuals without hearing complaints seek health care, when compared to those who do not study, or those who receive health care may be getting some counseling/treatment for hearing loss prevention. This can also be explained by the fact that individuals may be receiving information on hearing loss in the elderly from other professionals. Additionally, elderly individuals may be experiencing conditions that are common in the assessed population and may have hearing loss as a consequence, such as diabetes, hypertension, dyslipidemia, among others.

Pandhi et al.^17^ investigated whether the tendency of elderly individuals in reporting difficulties, delays and decreased satisfaction in access to health care would be related to hearing loss. They found that individuals with hearing impairment were more likely to report difficulties in access to health care; however, hearing was not a predictive factor of satisfaction with healthcare access.

We found a significant association between social activity, including physical activity, and hearing loss in the elderly. Another study showed no significant association between physical activity and hearing loss complaints.^18^

Corroborating the findings of our study, Chen et al.^19^ in their research also found that hearing loss in the elderly is independently associated with increased impairment and limitations in several categories of self-reported physical functioning. In contrast, the study by Fieldler and Peres^20^ emphasizes that there seems to be an association between the increased incidence of hearing loss and that of physical activity practice, allowing us to reflect that individuals who perform physical activity may have more complaints, as they become annoyed when they cannot interact with their surrounding environment.

About the self-reported ethnicity (white, mixed-race, black, or other) and the association with hearing complaints, the difference was not statistically significant between the groups in our study. However, a greater tendency of complaints was reported among blacks, followed by mixed-race and white individuals. We must emphasize the fact that the proportion of non-white individuals is much smaller in the state of Rio Grande do Sul when compared to other states of Brazil, with this fact being a reason for not achieving the recommended level of significance. This finding differs from those found in a North-American study, in which the black ethnicity in the elderly was considered significantly protective against hearing loss.^21^

We found that older individuals who self-report hearing complaints prefer not leaving the house due to communication difficulties, but a similar study did not show the same results. It analyzed social isolation and its association with hearing loss and found that a greater degree of hearing loss was associated with increased chances of social isolation only in women aged 60–69 years, whereas this association was not significant in other age ranges and in men.^22^ Individuals who did not leave home due to communication difficulties had a 95% higher chance of having hearing complaints, according to our findings. It is suggested that hearing loss limits communication and brings significant restrictions to the elderly when leaving home, although this was not the only cause.

We observed that only one-third of the individuals who used a hearing aid did not mention hearing complaints, thus inferring that the hearing aid fully meets their hearing needs. Lack of adjustments or inadequacies could explain the percentage of individuals with hearing complaints even while using a hearing aid.

While the numbers of this study enlighten us about the issues regarding hearing loss, it is clear that very little has been done about it. Among our population, we found that only four subjects received audiology follow-up and 54 used hearing aids. Also, we suggest a population-based study with an instrumental hearing assessment, since it is believed that the number of elderly with complaints is lower than the number of those who actually have some degree of hearing loss, considering that hearing loss is progressive (making it easier for the elderly to adapt to the loss), that few of them perform social activities (thus, not being exposed to environments that require good hearing) and that most do not receive health care monitoring.

We emphasize the importance of implementing longitudinal follow-up studies of elderly patients with hearing loss complaints, so that we can establish different treatment options, aimed not only at the treatment, but also at more effective prevention in this ever-growing population, especially regarding its clinical, physical, functional and psychosocial aspects.

## Conclusion

Our study clearly shows that there is a prevalence of approximately 30% of hearing loss complaints among the elderly. The hearing complaint is observed more frequently in men with fewer years of schooling. The chance of having hearing complaints increases with age: for every extra year of age there is an increase of 6% in the chance of hearing loss complaint. The complaint was associated with decreased access to health care and not leaving the house due to communication difficulties. On the other hand, elderly individuals with higher levels of social activity had a higher frequency of hearing complaints.

## Conflicts of interest

The authors declare no conflicts of interest.
